# Characterization of the *Lycium barbarum* fruit transcriptome and development of EST-SSR markers

**DOI:** 10.1371/journal.pone.0187738

**Published:** 2017-11-10

**Authors:** Chunling Chen, Meilong Xu, Cuiping Wang, Gaixia Qiao, Wenwen Wang, Zhaoyun Tan, Tiantian Wu, Zhengsheng Zhang

**Affiliations:** 1 Engineering Research Center of South Upland Agriculture, Ministry of Education, Southwest University, Chongqing, China; 2 State Key Laboratory of Seedling Bioengineering, Ningxia Forestry Institute, Yinchuan, China; Chinese Academy of Agricultural Sciences Cotton Research Institute, CHINA

## Abstract

*Lycium barbarum*, commonly known as goji, is important in Chinese herbal medicine and its fruit is a very important agricultural and biological product. However, the molecular mechanism of formation of its fruit and associated medicinal and nutritional components is unexplored. Moreover, this species lacks SSR markers due to lack of genomic and transcriptomic information. In this study, a total of 139,333 unigenes with average length of 1049 bp and N50 of 1579 bp are obtained by trinity assembly from Illumina sequencing reads. A total of 92,498 (66.38%) unigenes showed similarities in at least one database including Nr (46.15%), Nt (56.56%), KO (15.56%), Swiss-prot (33.34%), Pfam (33.43%), GO (33.62%) and KOG/COG (17.55%). Genes in flavonoid and taurine biosynthesis pathways were found and validated by RT-qPCR. A total of 50,093 EST-SSRs were identified from 38,922 unigenes, and 22,537 EST-SSR primer pairs were designed. Four hundred pairs of SSR markers were randomly selected to validate assembly quality, of which 352 (88%) were successful in PCR amplification of genomic DNA from 11 *Lycium* accessions and 210 produced polymorphisms. The polymorphic loci showed that the genetic similarity of the 11 *Lycium* accessions ranged from 0.50 to 0.99 and the accessions could be divided into 4 groups. These results will facilitate investigations of the molecular mechanism of formation of *L*. *barbarum* fruit and associated medicinal and nutritional components, and will be of value to novel gene discovery and functional genomic studies. The EST-SSR markers will be useful for genetic diversity evaluation, genetic mapping and marker-assisted breeding.

## Introduction

*Lycium barbarum* belongs to the *Lycium* genus, which is widely distributed in northwest China and has been used as a traditional herbal medicine for thousands of years. The fruit of *Lycium barbarum* have a variety of pharmacologic and hygienic functions [1–11]. Since the beginning of this century, the fruits and juice of *L*. *barbarum* have been sold as health food products and praised in advertisements and in the media for well-being and as an anti-aging remedy [12]. Recently, *L*. *barbarum* has become a leading commercial crop in some areas of China.

In the last few years, many breeding scientists have invested much effort to breed *L*. *barbarum* cutivars with high fruit yield and quality, but it is hard and usually takes many years to develop a new cultivar with stably inherited target characters because the species is perennial. New technologies can accelerate breeding through improving genotyping and phenotyping methods and increasing the available genetic diversity in breeding germplasm [13]. However, there are few genomic resources in *L*. *barbarum*. With the development of massively-parallel (‘next generation’) sequencing, we can rapidly sequence the transcriptome of an organism by the ‘RNA-seq’ approach, which is essential for interpreting functional elements of the genome and revealing the molecular constituents of cells and tissues [14], and RNA-seq is also a very good way to develop EST-SSR markers. Recently, transcriptome studies and functional gene mining by RNA-seq were reported in many species which have no genome sequencing, such as *Piper nigrum* [15], *Litchi chinensis* Sonn [16], *Arceuthobium sichuanense* [17], *Idesia polycarpa* [18], *Cinnamomum camphora* L [19], and *Lycium chinense* Mill, a relative of *L*. *barbarum* [20].

Marker-assisted selection offers great potential to improve the efficiency of breeding perennials. SSR markers are particularly useful for a variety of applications in plant genetics and breeding because of their reproducibility, multiple alleles, codominant inheritance, relative abundance and good genome coverage [21]. SSR markers can be developed directly from random genomic DNA libraries or from libraries enriched for specific microsatellites. For those species lacking sequenced genomes and/or rich expressed sequence tag (EST) resources, transcriptome scans from RNA-seq offer a means to develop SSR markers. Recently the development of EST-SSR markers from RNA-seq based transcriptomes has been reported in *Juglans mandshurica* [22], *Lindera glauca* [23], *Caragana korshinskii Kom* [24], *Camellia sinensis* [25], radish [26], and others.

There are only a few reports of the use of molecular markers in *L*. *barbarum*. Zhang et al. distinguished *L*. *barbarum* from other closely related species by RAPD techniques [27]. Kwon et al. isolated and characterized 21 polymorphic microsatellite markers in *L*. *chinense*, a relative of *L*. *barbarum* [28]. Subsequently, Zhang et al. assessed the genetic diversity and population structure of 139 *L*. *chinense* accessions with 18 of the 21 polymorphic *L*. *chinense* SSR markers [29]. However, there is no report of the development and use of SSR markers in *L*. *barbarum*.

The fruit of *L*. *barbarum* is a very important agricultural and biological product with medicinal and nutritional properties. In this study, by transcriptome sequencing of *L*. *barbarum* fruit, we aimed to provide a resource for functional gene mining, and develop EST-SSR markers which can be used for genetic diversity evaluation, construction of linkage maps, fine mapping of crucial genes and marker-assisted breeding. This study will provide useful information to better understand the molecular mechanism of *L*. *barbarum* fruit development.

## Material and methods

### Sample collection, RNA and DNA extraction

For transcriptomic sequencing, fruit of 5 days, 15 days and 30 days after flowering and different tissues (root, stem, leaf) were collected from a 5-year-old *L*. *barbarum* (Ningqi1) tree growing in the germplasm nursery of the Ningxia Forestry Institution in China in July, 2016. To verify polymorphism of EST-SSR markers for subsequent population genetic studies, leaf samples were collected from 8 *L*. *barbarum* accessions, a Korea wolfberry accession, a black fruit wolfberry (*L*. *ruthenicum*) accession, and a Big leaf wolfberry (*L*. *chinense*) accession (Table 1) in the same germplasm nursery in 2016. All samples were frozen immediately in liquid nitrogen and stored at −80 C.

**Table 1:** *Lycium* accessions used for EST-SSR verification.

Number	Accessions or cultivars name	Species	Source (GPS)
1	Ningqi 1	*Lycium barbarum* L.	Ningxia, China (38°47'N, E106°27')
2	Ningqi 3	*Lycium barbarum* L.	Ningxia, China (38°47'N, E106°27')
3	Ningqi 4	*Lycium barbarum* L.	Ningxia, China (38°47'N, E106°27')
4	Ningqi 5	*Lycium barbarum* L.	Ningxia, China (38°47'N, E106°27')
5	Ningqi 6	*Lycium barbarum* L.	Ningxia, China (38°47'N, E106°27')
6	Ningqi 7	*Lycium barbarum* L.	Ningxia, China (38°47'N, E106°27')
7	Ningqi 8	*Lycium barbarum* L.	Ningxia, China (38°47'N, E106°27')
8	Ningqi 9	*Lycium barbarum* L.	Ningxia, China (38°47'N, E106°27')
9	Korea wolfberry	—	Korea (37°33'N, 126°58'E)
10	Black fruitwolfberry	*Lycium ruthenicum* Murr	Gansu, China (38°93'N, 100°46'E)
11	Big leaf wolfberry	*Lycium chinense* Mill	Guangdong, China (23°12'N, 113°28 'E)

The fruit and tissue samples of Ningqi1 were ground to a powder in liquid nitrogen and total RNA was extracted using TaKaRa MiniBEST Universal RNA Extraction Kit. RNA degradation and contamination was monitored on 1% agarose gels. RNA purity was checked using the NanoPhotometer^®^ spectrophotometer (IMPLEN, CA, USA). RNA concentration was measured using the Qubit^®^ RNA Assay Kit in Qubit^®^ 2.0 Fluorometer (Life Technologies, CA, USA). RNA integrity was assessed using the RNA Nano 6000 Assay Kit of the Agilent Bioanalyzer 2100 system (Agilent Technologies, CA, USA). Genomic DNA was extracted from young leaves of the 11 *Lycium* accessions using a modified CTAB method [30]. DNA was resuspended in 50 μL of water and dilutions were performed to obtain a final concentration of 10 ng/μL and stored at −20°C until use.

### Library preparation for transcriptome sequencing

A total of 1.5 μg RNA per sample was used as input material for RNA sample preparations. Sequencing libraries were generated using NEBNext^®^ Ultra^™^ RNA Library Prep Kit for Illumina^®^ (NEB, USA) following the manufacturer’s recommendations and index codes were added to attribute sequences to each sample. Briefly, mRNA was purified from total RNA using poly-T oligo-attached magnetic beads. Fragmentation was carried out using divalent cations under elevated temperature in NEBNext First Strand Synthesis Reaction Buffer (5×). First strand cDNA was synthesized using random hexamer primers and M-MuLV Reverse Transcriptase (RNase H). Second strand cDNA synthesis was subsequently performed using DNA Polymerase I and RNase H. Remaining overhangs were converted into blunt ends via exonuclease/polymerase activities. After adenylation of 3’ ends of DNA fragments, NEBNext Adaptors with hairpin loop structure were ligated to prepare for hybridization. In order to select cDNA fragments of 150~200 bp in length, the library fragments were purified with the AMPure XP system (Beckman Coulter, Beverly, USA). Then 3 μl USER Enzyme (NEB, USA) was used with size-selected, adaptor-ligated cDNA at 37°C for 15 min followed by 5 min at 95°C before PCR. PCR was performed with Phusion High-Fidelity DNA polymerase, Universal PCR primers and Index (X) Primer. PCR products were purified (AMPure XP system) and library quality was assessed on the Agilent Bioanalyzer 2100 system.

### Sequencing and transcriptome assembly

Clustering of the index-coded samples was performed on a cBot Cluster Generation System using TruSeq PE Cluster Kit v3-cBot-HS (Illumina) according to the manufacturer’s instructions. After cluster generation, the libraries were sequenced on an Illumina HiSeq 4000 platform and paired-end reads were generated. *De novo* transcriptome assembly was accomplished using trinity (r20140413p1) with default settings [31].

### Gene function annotation

Unigenes of the transcriptome were annotated based on data from the Nr (NCBI non-redundant protein sequences), Nt (NCBI non-redundant nucleotide sequences), Pfam (Protein family), KOG/COG (Clusters of Orthologous Groups of proteins), Swiss-Prot (manually annotated and reviewed protein sequence), KO (KEGG Ortholog), and GO (Gene Ontology) databases. To further analyze the transcriptome of *L*. *barbarum*, all unigenes were submitted to the KEGG pathway database. All BLAST searches were performed with an e-value of 1E^-5^.

### Analysis of unigenes related to flavonoid biosynthesis and taurine biosynthesis

*L*. *barbarum* cDNA was generated using TaKaRa Prime Script TM RT reagent Kit with gDNA Eraser(Perfect Real Time) from the extracted RNA of fruits collected 5 days, 15 days and 30 days after flowering and different tissues (root, stem and leaf) of *L*. *barbarum*. Then qRT-PCR was performed to analyze the relative expression for the genes *LbCHI*, *LbC4H*, *LbDFR*, *LbANR*, *LbANS*, *LbFLS*, *LbLAR*, *LbF3H* and *LbCDO-like* by SYBR Premix Ex Taq II (Tli RNaseH Plus) in qTOWER2.2 REAL-TIME PCR Thermal Cycler (analytikjena biometra). Specific primers were listed in S1 Table.

### Development and detection of EST-SSR markers

SSRs in the transcriptome were identified using the microsatellite identification tool MISA (http://pgrc.ipkgatersleben.de/misa/misa.html), and primers for each SSR designed using Primer 3 (http://primer3.sourceforge.net/releases.php) according to the following parameters: length range from 18 to 23 nucleotides with 20 bp as optimum, PCR product size range from 100 to 300 bp, optimum annealing temperature from 55°C~60°C, and GC content 40–60% with 50% as optimum. In total, 400 primer pairs (S2 Table) were randomly selected to evaluate amplification and polymorphism in L. barbarum. PCR amplification was performed on a Veriti^®^ 96-Well Thermal Cycler using the following thermal profile: 94°C for 5 min; 35 cycles of 94°C for 30 s, 55°C for 30 s and 72°C for 2 min; then extension of products at 72°C for 10 min. The PCR products were separated by electrophoresis on 8.0% non-denaturing polyacrylamide gels, silver-stained, and band sizes assessed by comparison to a DNA ladder.

## Results

### Illumina sequencing and *de novo* assembly

cDNA was prepared from 5 days, 15 days, and 30 days fruits after flowering and sequenced with Illumina HiSeq 4000 platform. A total of 46,486,152, 49,649,472, 56,409,052 raw reads were generated, after stringent quality assessment and data filtering, a total of 44,190,154, 47,458,054, 53,607,998 clean reads were generated for 5 days, 15 days, and 30 days fruits. All high-quality reads were assembled using trinity software [31], yielding a total of 219,831 transcripts with average length of 771 bp and N50 of 1302 bp (Table 2). The length distribution of transcripts is showing in S1 Fig. The de novo assembled transcriptomes were clustered by ‘corset’, which is a method and software for obtaining gene-level counts from any de novo transcriptome assembly [32]. After clustering by corset, a total of 139,863 clusters were obtained, in which the clusters with longest sequences were defined as unigenes. Finally a total of 139,333 unigenes with average length of 1049 bp and N50 of 1579 bp (Table 2) were obtained from the transcripts. The length distribution of unigenes is showing in S2 Fig. The length distribution comparison of transcripts and unigenes is showing in S3 Fig.

**Table 2:** Characteristics of assembled transcripts and unigenes.

Nucleotide length (bp)	Transcripts	Unigenes
200–500	125,433	47,372
501–1,000	46,113	43,757
1,001–1,500	18,290	18,213
1,501–2,000	11,326	11,322
2,001–2,500	7,184	7,195
2,501–3,000	4,576	4,565
>3,000	6,909	6,909
Total	219,831	139,333
N50 (bp)	1,302	1,579
Average length (bp)	771	1,049
Min length (bp)	201	201
Median length (bp)	426	687
Max length (bp)	15,884	15,884
Total nucleotide length (bp)	169,512,437	146,170,451

### Functional annotation of unigenes

**Fig 1:**
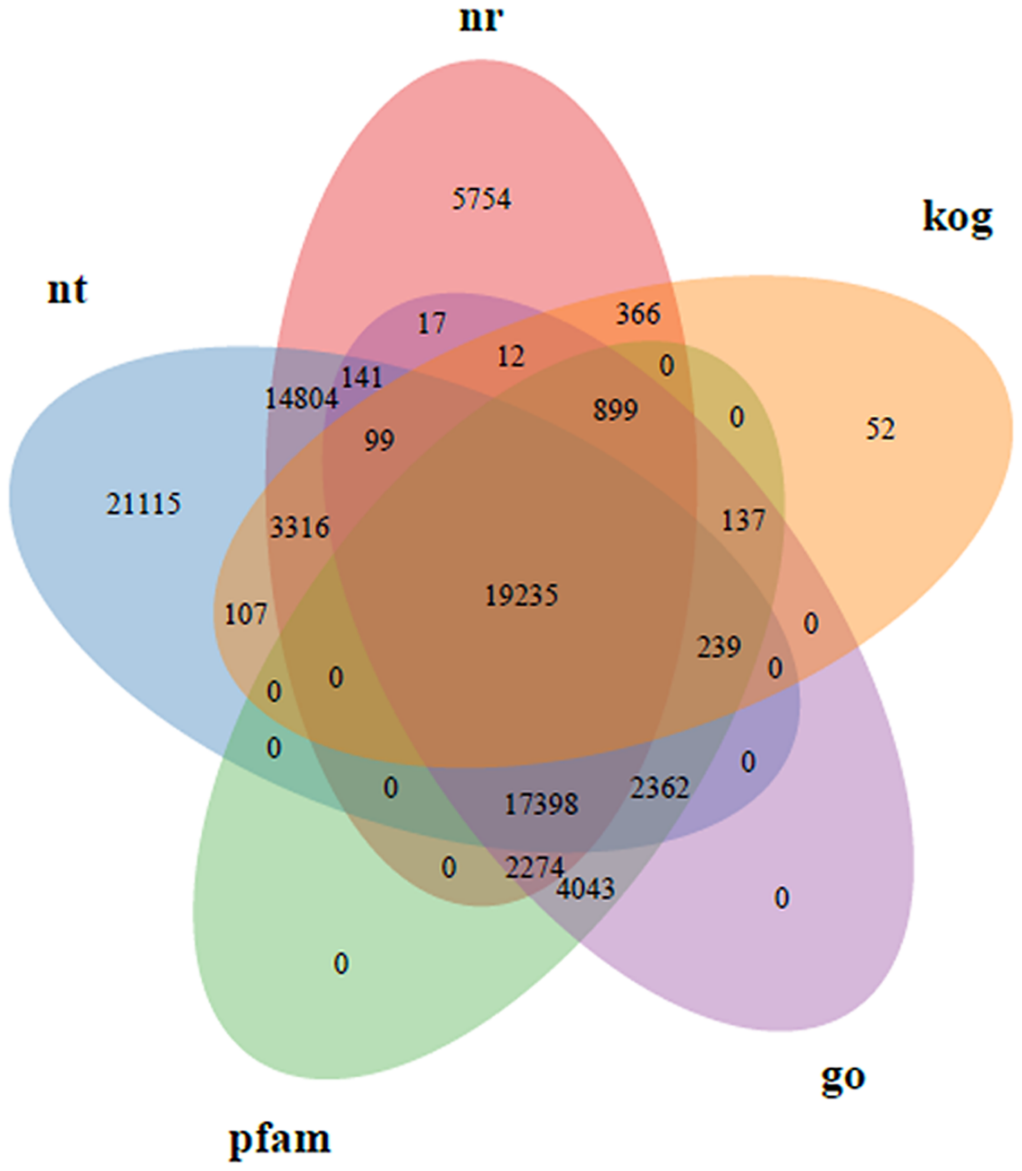
Functional annotation of the *Lycium barbarum L*. transcriptome.

To validate the assembly quality and annotation of the assembled unigenes, all unigenes were used to seek matches in public databases including Nr, Nt, Ko, Swiss-prot, Pfam, GO and KOG/COG using the BLASTx program with an E-value threshold of 1E^-5^. Among 139,333 unigenes, a total of 12,246 (8.78%) unigenes were annotated in all databases, and 92,498 (66.38%) matched genes and/or proteins in at least one database. The detailed results are shown in Fig 1 and S3 Table.

**Fig 2:**
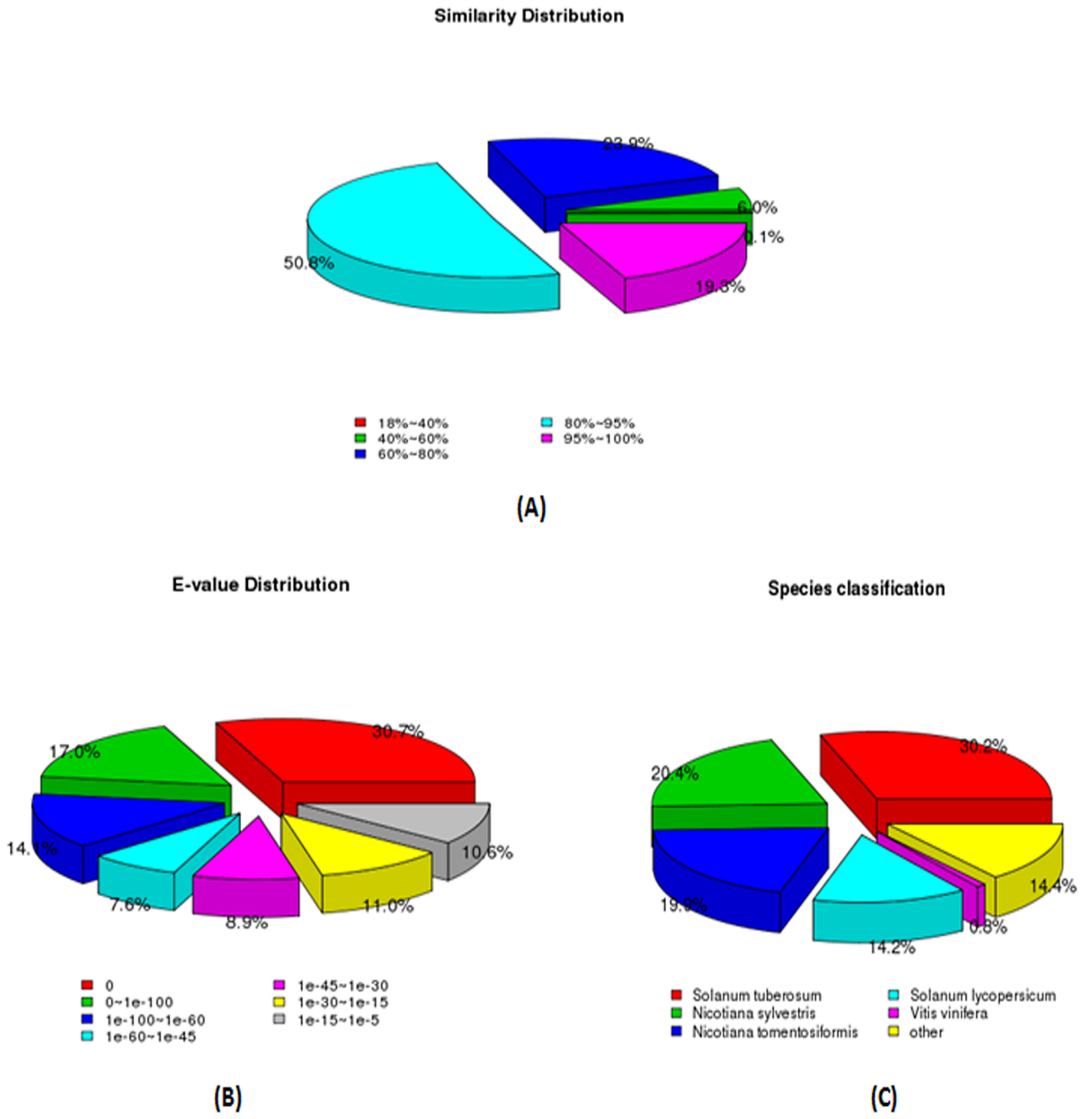
Characterization of assembled *Lycium barbarum* unigenes using the Nr databases. (A) Similarity distribution of the top BLAST hits for the assembled unigenes with a cutoff of 1E^-5^. (B) E-value distribution of BLAST hits for the assembled unigenes with a cutoff of 1E^-5^. (C) Species distribution of the top BLAST hits for the assembled unigenes.

Based on the Nr database, of the assembled sequences, 66.76% showed significant homology (<1E-50), and 70.09% showed more than 80% similarity to Nr database entries (Fig 2A and 2B). The *L*. *barbarum* unigenes were homologous to sequences in other species, among which *Solanum tuberosum* accounted for 30.2% (19,388), *Nicotiana sylvestris* accounted for 20.4% (13138), *Nicotiana tomentosiformis* accounted for 19.9% (12,793), *Solanum lycopersicum* accounted for 14.2% (9112), *Vitis vinifera* accounted for 0.8% (542), and others 14.4% (9276) (Fig 2C).

**Fig 3:**
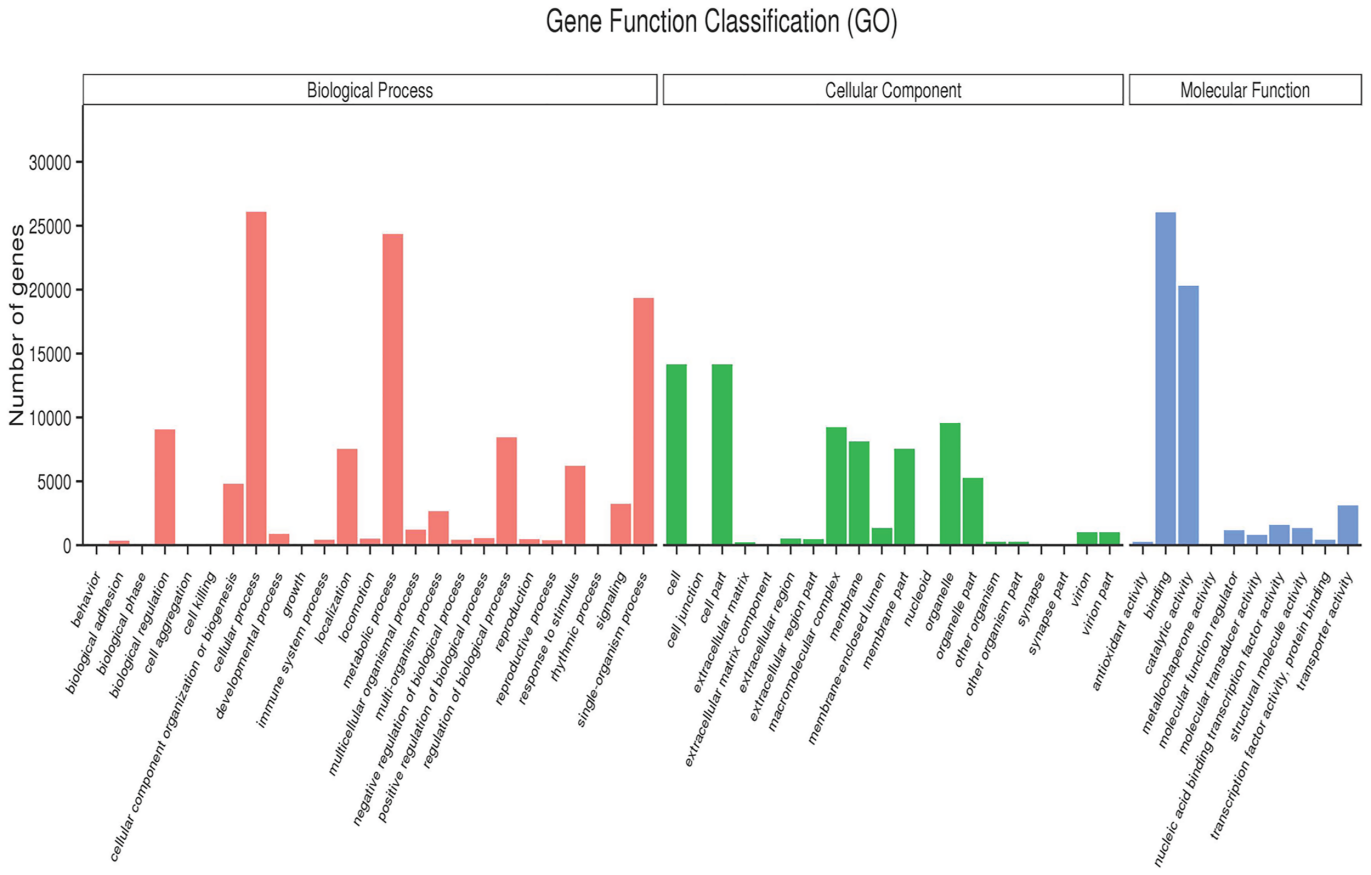
Gene Ontology classifications of assembled unigenes.

Based on the Nr annotation, then we used GO analysis to classify functions and understand the general distribution of the unigenes of *L*. *barbarum*. In the present study, 46,856 unigenes matching known protein databases were assigned to 55 GO functional groups with 245,532 functional terms. As shown in Fig 3 and S4 Table, assignments to biological process are the majority (117,337, 47.79%), followed by cellular component (73,101, 29.77%) and molecular function (55,094, 22.44%). Under the biological process category, “cellular process” (26,093, 22.24%) and “metabolic process” (24,355, 20.76%) were represented prominently. In the cellular component, “cell”, “cell part”and “organelle” accounted for 96.64%, however, there are a few unigenes in the “extracellular region part”, “virion” and “virion part”. Under the classification of molecular function, “binding”(26,067, 47.31%) is the largest category and 8370 unigenes in “antioxidant”, “structural molecule”, “transporter molecule”, “transducer activity”, and “molecular function regulator” only accounted for 15.19%.

**Fig 4:**
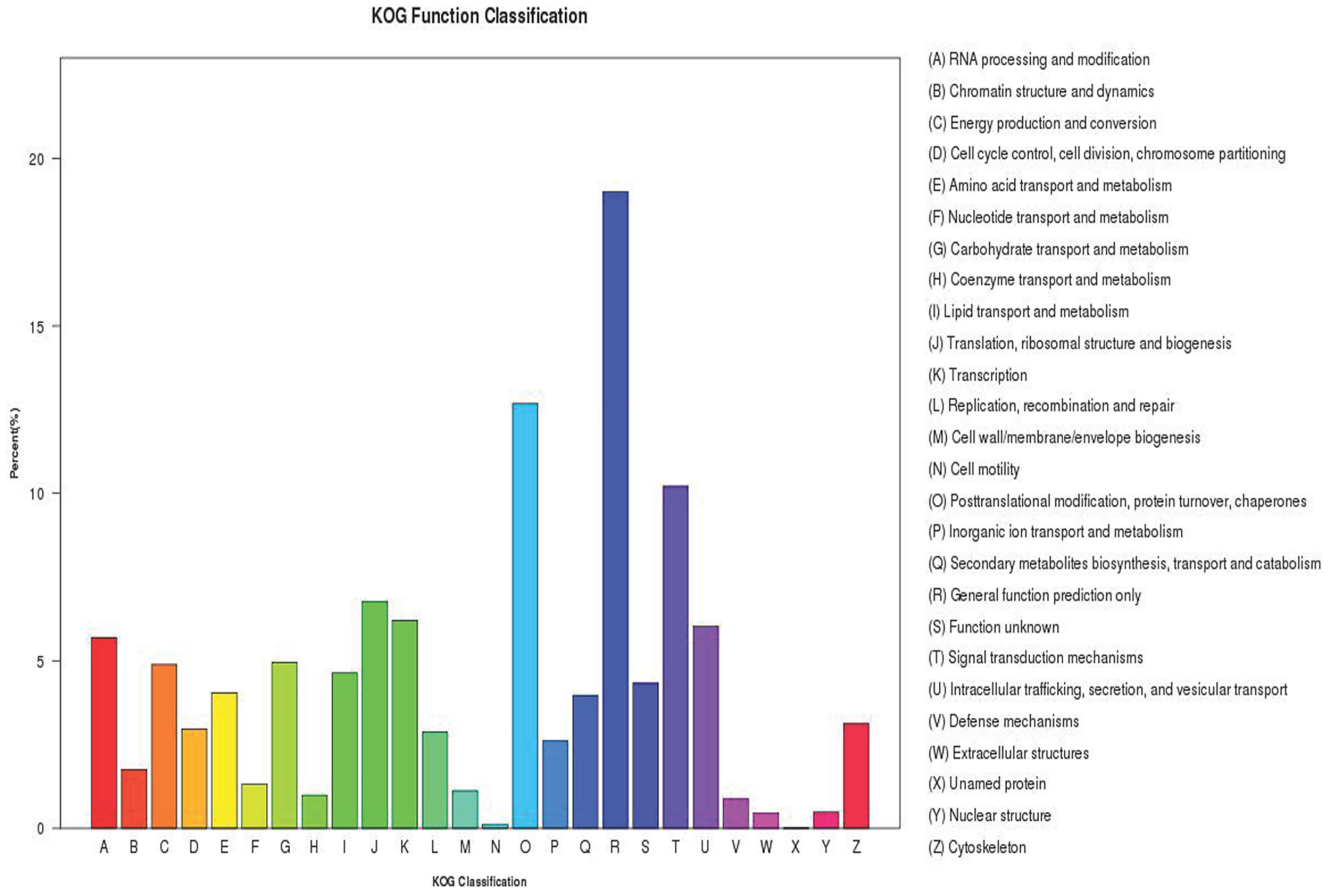
COG classification of *Lycium barbarum* fruit unigenes.

Among the 64,315 unigenes with similarity to Nr proteins, 24,462 were assigned to 26 COG classifications (Fig 4, S5 Table). Out of the 26 COG categories, the largest group is the cluster for “general function prediction”(4647, 16.95%), followed by “post-translational modification”, protein turnover and chaperones (3101, 11.31%); translation, ribosomal structure and biogenesis (1657, 6.04%); transcription (1518, 5.54%); Other categories including cell wall/membrane/envelope biogenesis, coenzyme transport and metabolism, cell motility defense mechanisms, extracellular structures, unamed proteins, and nuclear structure accounted for only less than 1% (Fig 4), was in the smallest group.

**Fig 5:**
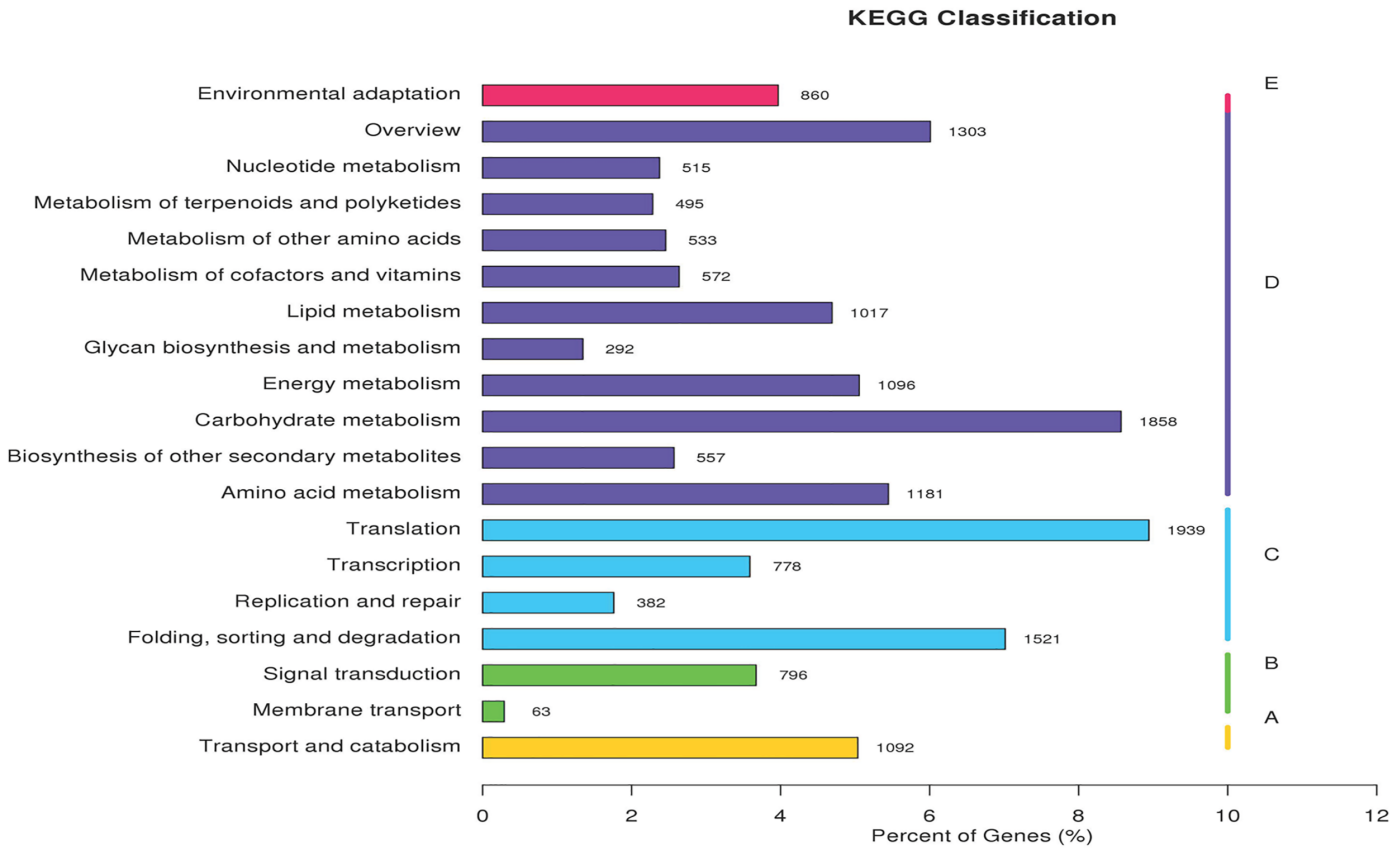
KEGG classification of *Lycium barbarum* fruit unigenes.

To further investigate the functions of *L*. *barbarum* fruit unigenes, the KEGG pathway database was used. Among the 21,684 unigenes, 16,850 (77.71%) were classified into 5 main categories (Fig 5, S6 Table) including 123 KEGG pathways. “Metabolism” was the biggest category (9419, 55.90%), followed by “genetic information processing” (4,620, 27.42%), “cellular processes” (1092, 6.48%), “organismal systems” (860, 5.10%) and “environmental information processing” (859, 5.10%). A total of 11 categories are contained in the KEGG metabolism, such as “carbohydrate metabolism”, “nucleotide metabolism”, “amino acid metabolism”, “lipid metabolism”, “energy metabolism”, and the “biosynthesis of other secondary metabolism”.

*L*. *barbarum* fruit have high pharmacological and hygienic function components, which usually come from secondary metabolites. We found 269 unigenes related to other secondary metabolites in the transcriptome of *L*. *barbarum* fruit (S6 Table) encoding genes involved in anthocyanin biosynthesis (13), betalain biosynthesis (5), flavone and flavonol biosynthesis (23), and flavonoid biosynthesis (59).

Fruit of *L*. *barbarum* are rich in amino acids, which are an important element of their nutritional value. There are 2155 unigenes encoding amino acid metabolism and biosynthesis in the *L*. *barbarum* fruit transcriptome (Table 3), encoding arginine biosynthesis (92); lysine biosynthesis (19); phenylalanine, tyrosine and tryptophan biosynthesis (111); valine, leucine. and isoleucine biosynthesis (65); taurine and hypotaurine metabolism(15).

**Table 3:** Correspondence of *Lycium barbarum* fruit unigenes to pathways involved in amino acid metabolism.

KEGG Pathway	Pathway ID	Gene Number
Alanine, aspartate and glutamate metabolism	ko00250	136
Arginine and proline metabolism	ko00330	137
Arginine biosynthesis	ko00220	92
Cysteine and methionine metabolism	ko00270	219
Glycine, serine and threonine metabolism	ko00260	187
Histidine metabolism	ko00340	50
Lysine biosynthesis	ko00300	19
Lysine degradation	ko00310	93
Phenylalanine metabolism	ko00360	88
Phenylalanine, tyrosine and tryptophan biosynthesis	ko00400	111
Tryptophan metabolism	ko00380	77
Tyrosine metabolism	ko00350	131
Valine, leucine and isoleucine biosynthesis	ko00290	65
Valine, leucine and isoleucine degradation	ko00280	178
Cyanoamino acid metabolism	ko00460	131
Glutathione metabolism	ko00480	214
Selenocompound metabolism	ko00450	68
Taurine and hypotaurine metabolism	ko00430	15
beta-Alanine metabolism	ko00410	144

### Analyzing unigenes related to flavonoid biosynthesis

**Fig 6:**
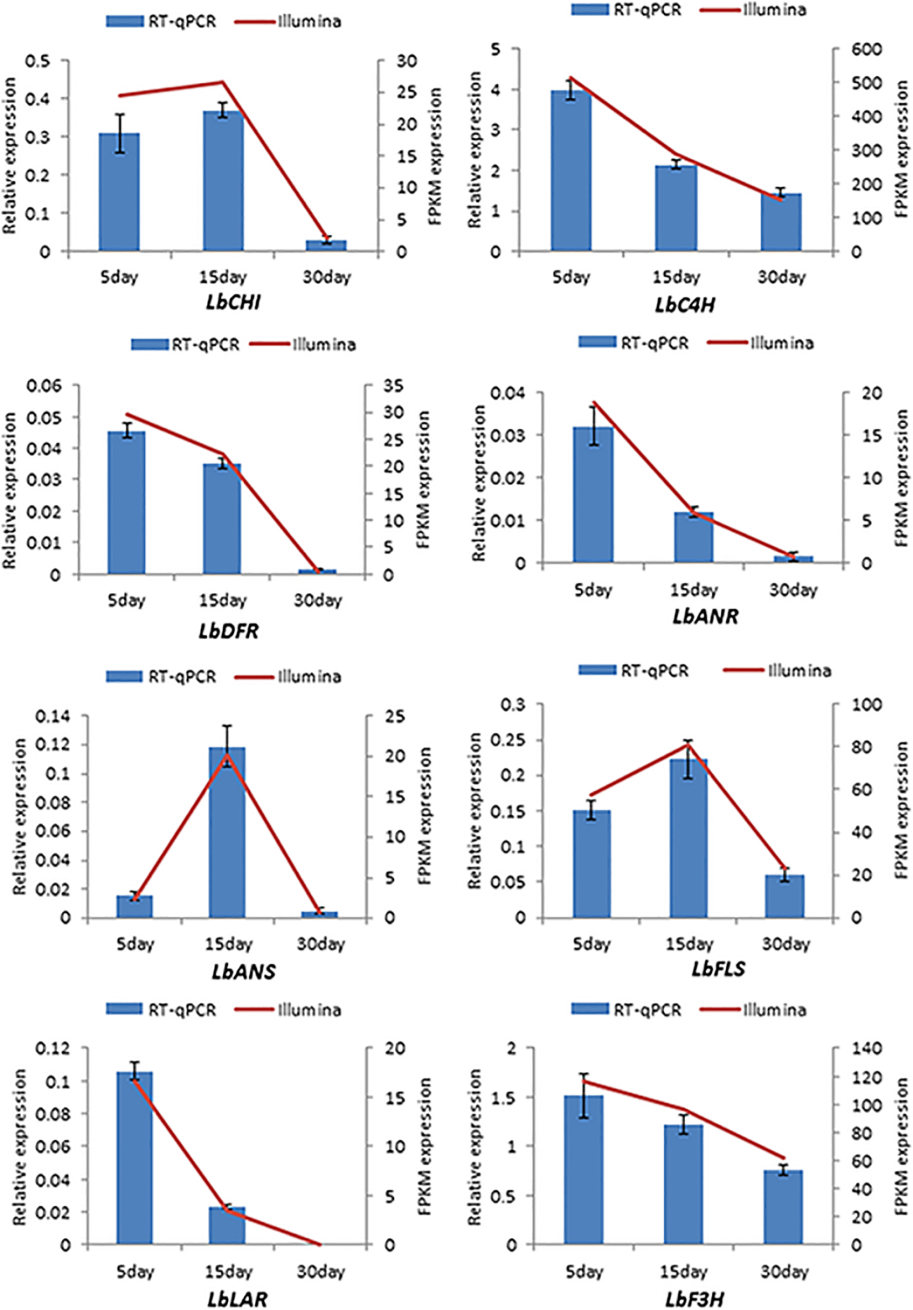
RT-qPCR validation of selected unigenes involved in triterpene flavonoid biosynthesis.

To confirm the accuracy of the sequencing, assembly and annotation results, 8 important genes in the pathway of flavonoid biosynthesis including chalcone isomerase (CHI), cinnamate 4-hydroxylase (C4H), dihydro flavonol 4-reductase (DFR), anthocyanidin reductase (ANR), anthocyanidin synthase (ANS), flavonol synthase (FLS), leucoanthocyanidin reductase (LAR), flavanone 3-hydroxylase (F3H) were selected to determine their relative expression level in different stages of fruit development by RT-qPCR. The RT-qPCR and FPKM results were compared and the results are presented in Fig 6, The expression levels of the 8 genes obtained by RT-qPCR and the FPKM calculation showed the same trend of expression in different stages of fruit development, indicating the accuracy of transcriptome sequencing, assembly and functional annotation of unigenes of the *L*. *barbarum* fruit.

### Analyzing unigenes related to taurine biosynthesis

**Fig 7:**
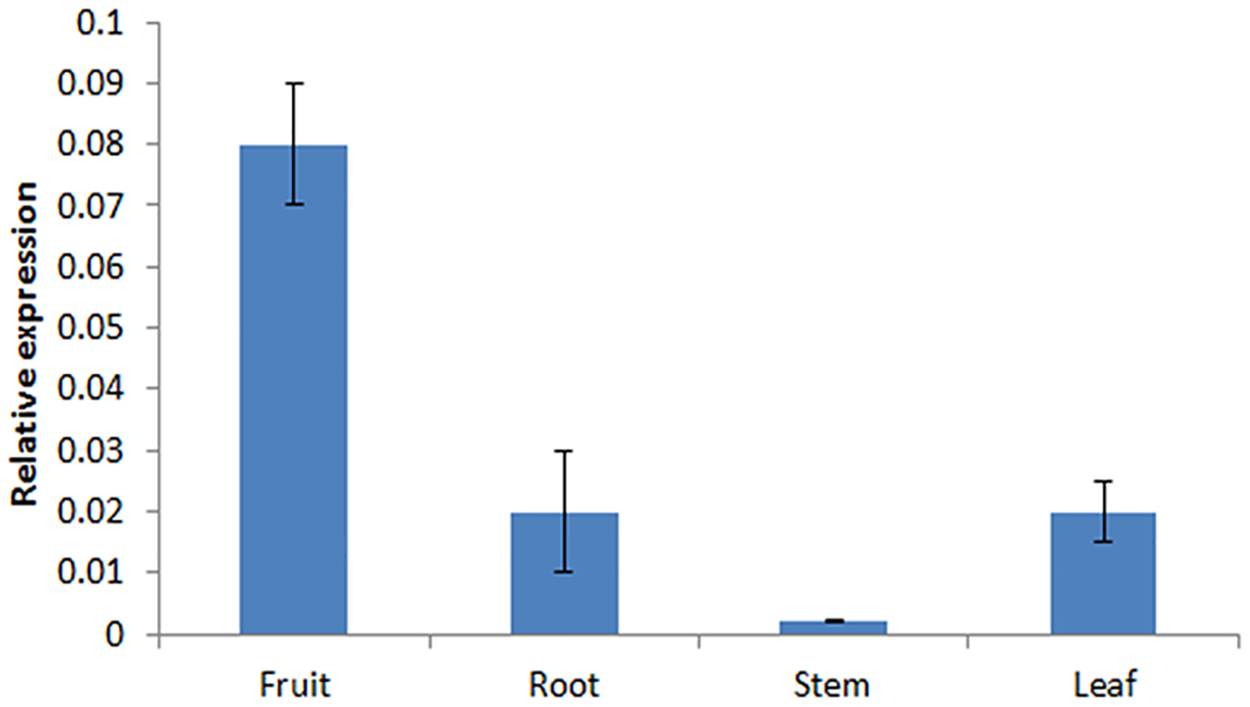
Relative expression of an *LbCDO-like* gene in different tissues of *Lycium barbarum*.

Among all the amino acids in the fruit of *L*. *barbarum*, taurine is a special pharmacologically and hygienic functional component. From the functional classification by KEGG (Table 3), 15 genes encoding taurine and hypotaurine metabolism were found from the transcriptome of *L*. *barbarum* fruit. Among the 15 genes, one was expressed highly in different stages of fruit development and annotated as cysteamine dioxygenase (CDO), which is the crucial enzyme of taurine biosynthesis. To validate that the *CDO-like* gene isexpressed in the fruit of *L*. *barbarum*, the relative expression level of the *CDO-like* gene in different tissues (fruit, root, stem and leaf) was detected by RT-qPCR. We can see the result from Fig 7 the *LbCDO-like* gene was expressed at a high level in the ripening fruit compared to the root, stem, and leaf, indicating that it may play an important role in fruit ripening, which may contribute to taurine biosynthesis and accumulation in the fruit of *Lycium barbarum*.

### Development and characterization of EST-SSR markers

To develop new molecular markers, the 139,333 unigenes generated in this study were used to mine potential microsatellites using MISA soft (MISA, http://pgrc.ipk-gatersleben.de/misa/misa.html). A total of 50,093 EST-SSRs were identified from 38,922 unigenes, and 8,763 contained more than one SSR (Table 4). The EST-SSR frequency in the transcriptome was 35.95%, and the distribution density was 342.70 per Mb. Of the 50,093 SSRs, 33,013 are only one nucleotide with at least 10 repeats and 10, SSRs are more than one repeat motif, mostly di-nucleotide (51.82%), followed by tri-nucleotide (45.55%), tetra-nucleotide (2.23%), hexa-nucleotide (0.23%), and penta-nucleotide (0.16%) repeat units (Table 5). SSRs with six tandem repeats (29.26%) were the most common, followed by five (28.63%), seven (16.28%), eight (8.58%), nine (8.07%), ten (6.68%), and > 10 tandem repeats (2.49%). The dominant repeat motif in EST-SSRs was AG/CT (28.28%), followed by AT/AT (23.72%), AC/GT (11.36%), AAC/GTT (10.90%), and AAG/CTT (9.47%), AAT/ATT (5.43%) (Table 6), CG/CG (0.09%) was fewest.

**Table 4:** Summary of EST-SSRs identified in the *Lycium barbarum* L.transcriptome.

Searching item	Numbers
Total number of sequences examined	139,333
Total size of examined sequences (bp)	146,170,451
Total number of identified EST-SSRs	50,093
number of EST-SSRs containing more than one repeat motifs	10382
Number of EST-SSRs containing sequences	38922
Number of sequences containing more than one EST-SSRs	8763

**Table 5:** Frequency of EST-SSR repeat numbers in *Lycium barbarum* L.

Motif length	Repeat numbers							Total	%
	5	6	7	8	9	10	>10		
Dimer	—	1688	1072	843	836	694	247	5380	51.82
Trimer	2743	1316	613	47	2	—	8	4729	45.55
Tetramer	202	25	2	—	—	—	3	232	2.23
Pentamer	14	2	1	—	—	—	—	17	0.16
Hexamer	13	7	2	1	—	—	1	24	0.23
Total	2972	3038	1690	891	838	694	259	10382	
%	28.63	29.26	16.28	8.58	8.07	6.68	2.49		

**Table 6:** Frequency of di- and trinucleotide EST-SSR repeat motifs in *Lycium barbarum* L.

Repeat motif	Repeat numbers							Total	%
	5	6	7	8	9	10	>10		
AC/GT	—	168	119	99	55	37	16	494	11.36
AG/CT	—	369	264	182	194	151	70	1230	28.28
AT/AT	—	271	207	175	212	123	44	1032	23.72
CG/CG	—	3	1	—	—	—	—	4	0.09
AAG/CTT	206	190	71	—	1	—	6	474	10.90
AAC/GTT	209	131	68	4	—	—	—	412	9.47
AAT/ATT	98	80	53	5	—	—	—	236	5.43
ACC/GGT	73	22	6	—	—	—	—	101	2.32
ACG/CTG	15	12	1	—	—	—	—	28	0.64
ACT/ATG	63	20	10	5	—	—	—	98	2.25
AGC/CGT	21	7	2	4	—	—	—	34	0.78
AGG/CCT	45	12	7	2	—	—	—	66	1.52
AGT/ATC	68	24	16	2	—	—	—	110	2.53
CCG/CGG	22	8	1		—	—	—	31	0.71
Total	820	1317	826	478	462	311	136	4350	
%	18.85	30.28	18.99	10.99	10.62	7.15	3.13		

A total of 22,537 primer pairs were developed from the EST-SSR sites (S7 Table), and 400 (S2 Table) were randomly selected to evaluate their application and polymorphism in *L*. *barbarum* and other *Lycium* accessions (Table 1). Among the 400 primer pairs, 352 (88%) were successful in PCR amplification of genomic DNA from the 11 *Lycium* accessions, with 271 (76.99%) generating PCR products of the expected sizes, 81 (23.01%) generating larger than expected PCR products, and 205 with more than one band. A total of 210 pairs showed polymorphism and 451 polymorphic loci were detected in the 11 *Lycium* accessions. The number of loci per primer pair ranged from 1 to 9, with an average of 2.15.

**Fig 8:**
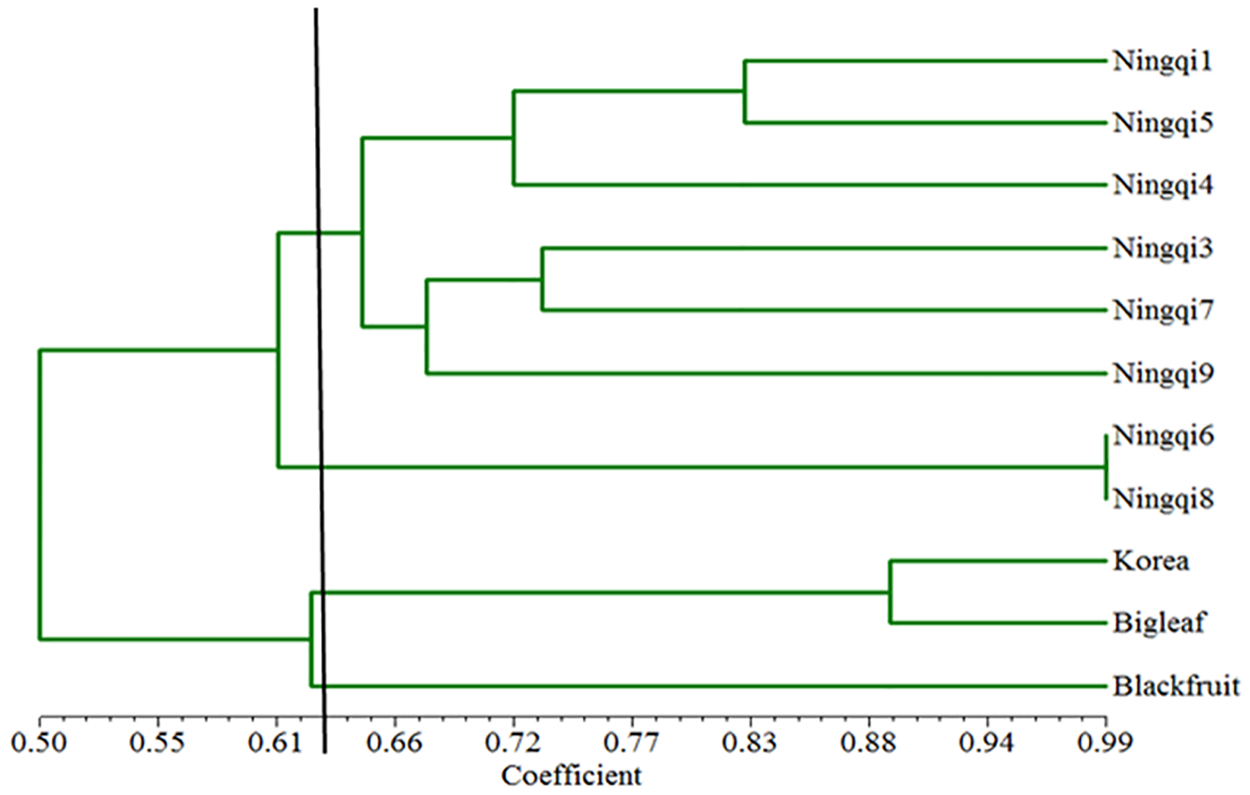
Dendrogram of 11 *Lycium* varieties based on EST-SSR markers.

All polymorphic loci were used to evaluate the genetic diversity and relationship among the 11 *Lycium* accessions. Genetic similarity of the 11 *Lycium* accessions (calculated by the NTSYS software) ranged from 0.50 to 0.99. Taking a genetic similarity score of 0.63 as the threshold, the 11 *Lycium* accessions could be divided into four groups (Fig 8). The first group includes black fruit wolfberry (*L*. *ruthenicum*), the second group includes big leaf wolfberry (*L*. *chinense*) and Korea wolfberry. The third group includes Ningqi6 and Ningqi8, and they have the highest genetic similarity (0.99). The fourth group includes Ningqi1, Ningqi3, Ningqi4, Ningqi5, Ningqi7 and Ningqi9.

## Discussion

### Characterization of the *L*. *barbarum* transcriptome

The high throughput and sensitivity of next-generation sequencing (NGS) has brought unprecedented opportunities for transcriptomic study. In contrast to microarray methods and Sanger sequencing of EST libraries, RNA sequencing (RNA-Seq) using NGS has many advantages in the characterization and quantification of transcriptomes. However, transcriptome assembly from billions of short reads poses a significant informatics challenge, which is also the bottleneck for the accuracy of the final result. There are many strategies and softwares for transcriptome assembly—for taxa lacking a reference genome, *de novo* assembly is usually the best choice. There are two methods of *de novo* assembly, based on overlap [33] such as CAP3 [34]; or on De-Bruijn graphs [35] which include velvet [36] ABySS [37], SOAP denovo [38], and Trinity [31]. Previous study indicated that overlap-layout-consensus (OLC) assemblers are well suited for very short reads and longer reads of small genomes respectively. For large datasets of more than hundreds of millions of short reads, De Bruijn graph-based assemblers would be more appropriate [39]. The use of an appropriate assembly tool for different species is very critical for the quality of the assembly, which is in turn critical to future analysis. In *L*. *barbarum*, a woody plant with a large genome, after Illumina HiSeq sequencing and removing reads containing adapter, poly-N or low quality sequence, clean reads with average length of 150 bp were used to assemble the transcriptome by Trinity software. A total of 139,333 unigenes were generated with an average length of 1049 bp and N50 of 1579 bp. The mean and N50 sizes of unigenes generated in the present study were obviously longer than those in the nearest relative with a transcriptome, *L*. *chinense* [20]. Indeed, the unigenes generated in this study (mean = 1049 bp) are longer than those assembled in other recent studies, for example, *Lonicera japonica* (882 bp) [40], *Arceuthobium sichuanense* (533 bp) [17], *Idesia polycarpa* (652 bp) [18], and *Cinnamomum camphora* (680 bp) [19]. These results suggest that the transcriptome sequencing data from *L*. *barbarum* fruit were effectively assembled.

### Functional annotation of unigenes

The *L*. *barbarum* fruit unigenes provide insight into the functions of genes active in fruit development, and which contribute to its medicinal and nutritional properties. Among 139,333 *L*. *barbarum* unigenes, 92,498 (66.38%) unigenes annotated in at least one database (among Nr, Nt, Ko, Swiss-prot, Pfam, GO and KOG/COG), the proportion of unigenes annotated is higher than that in *Arceuthobium sichuanense* (44.58%) [17], and *Idesia polycarpa* (48.2%) [18], which suggests that sequencing and assembly yielded unigenes with substantial functions. However, 33.62% of unigenes could not be matched to known proteins. Some of the unannotated unigenes are too short to have a characterized protein domain, whereas others with a known protein domain are highly diverged from other genes in the databases. Additionally, unannotated unigenes could derive from genes unique to *L*. *barbarum*, which contribute to its singular characteristics. The lack of a high quality *Lycium* genome limits the annotation resources available to further investigate unannotated unigene sequences.

The assembled unigenes represented a wide diversity of transcripts from *L*. *barbarum*, among which the KEGG pathways of biosynthesis of other secondary metabolism and amino acid metabolism were particularly important. The fruit of *L*. *barbarum* are rich in pharmacologically and hygienically active compounds such as anthocyanin, betalain, flavone, flavonoid, isoquinoline, tropane and others related to biosynthesis of secondary metabolites. The fruit also contains 17 amino acids [41]and taurine [42–44], which are the major bioactive constituents in the fruit. We found 269 unigenes encoding biosynthesis of secondary metabolites and 2155 unigenes encoding amino acid metabolism in the *L*. *barbarum* fruit transcriptome. This result provides a valuable resource for investigating specific processes, functions and pathways in the fruit of *L*. *barbarum*.

### Unigenes related to flavonoid and taurine biosynthesis

To confirm the accuracy of the sequencing, assembly and annotation results, 8 important genes in the pathway of flavonoid biosynthesis were selected to determine their relative expression level in different stages of fruit development by RT-qPCR and compared with the FPKM calculation. The results indicate the accuracy of transcriptome sequencing, assembly and functional annotation of unigenes of the *L*. *barbarum* fruit. This approach is widely used to valid the accuracy of transcriptome characterization[45–47] and is also a good way to mine the genes that we are interested in. Flavonoid in *L*. *barbarum* is a special pharmacologically and hygienic function component, which has the function of anti-cancer, anti-inflammation and anti-atherosclerosis [48,49]. Moreever, flavonoid biosynthesis is a metabolic pathway revealed early in different plants such as *Arabidopsis* [50], crop plants [51] and *Camellia sinensis* [52]. The study of flavonoid biosynthesis reflects well on the accuracy of gene mining in *L*. *barbarum*. In *L*. *barbarum*, there is no report about genes of flavonoid biosynthesis, and the genes found in this research will be conducive to promoting the study of flavonoid biosynthesis and metabolic mechanisms in *L*. *barbarum*.

Taurine is a free amino acid which is mainly present in animals, and has pharmacological and hygienic functions including effects on retinal development [53], antioxidation and neuroinhibition [54], treat of taurine deficiency retinopathy, kidney disease and congestive heart failure [55], and others. There are very few reports about taurine in plants except some seaweeds [56]. It is reported that taurine is abundant in the fruit of *L*. *barbarum* [42–44]. In this study, we found the taurine metabolic pathway from the transcriptome of *L*. *barbarum* fruit and one gene was annotated as cysteamine dioxygenase (CDO), which is the crucial enzyme of taurine biosynthesis [57]. This gene can express in different stages of fruit development and different tissues of *L*. *barbarum*, and is expressed at a high level in the ripening fruit compared to the root, stem, and leaf, indicating that it may contribute to taurine biosynthesis and accumulation in the fruit of *L*. *barbarum*. This is the first study about the genes for taurine metabolism in *L*. *barbarum*, The gene we found will provide a basis to support further molecular research on taurine biosynthesis in *L*. *barbarum*.

### EST-SSR marker characterization and validation

EST-SSR markers are of high value for research such as genetic diversity evaluation, construction of linkage maps, fine mapping of crucial genes and marker-assisted breeding. Because of the lack of a *L*. *barbarum* genome sequence, development of SSRs has been limited. In this study, numerous potential EST-SSR were identified from the *L*. *barbarum* transcriptome sequence. A total of 50,093 EST-SSRs were identified from 38,922 unigenes, and 22,537 primer pairs were designed from flanking sites. The EST-SSR frequency in the transcriptome was 35.95%, and the distribution density was 342.70 per Mb. This result indicates that there is a high frequency and distribution density of SSRs in the transcriptome of *L*. *barbarum*, higher than the reported in *Allium fistulosum* [58], and *Juglans mandshurica* [22]. Excluding mono-nucleotide repeats, the frequency of di-nucleotide was highest, followed by tri-nucleotide (45.55%), the same as in *Juglans mandshurica* [22] and *Caragana korshinskii* Kom [24]. The most abundant di-nucleotide motif was AG/CT, consistent with *Allium fistulosum* L.[58] and *Caragana korshinskii* Kom [24]. The most abundant tri-nucleotide motifs were AAG/CTT, consistent with *Camellia sinensis* [25] and radish [26].

Four hundred pairs of primers were randomly selected from the 22,537 EST-SSR markers to evaluate their application and the polymorphism rate in *L*. *barbarum* and other *Lycium* accessions. Among the 400 primer pairs, 352 (88%) were successful in PCR amplification with genomic DNA from 11 *Lycium* accessions, the remaining 12% either failing or producing only weak amplification, perhaps due to flanking a splice site resulting in large introns in the genomic sequence. Of the 352 primer pairs, 271 (76.99%) generated PCR products of expected size, while 81 (23.01%) were larger than expected, suggesting that the amplicons likely contained introns. A total of 205 pairs of primers generated PCR products with more than one band, that may result from the high heterozygosity and polyploidy of *L*. *barbarum* germplasm.

The 352 primers were used to analyze genetic relationships and diversity among 11 *Lycium* accessions. The 11 accessions were divided into 4 groups, with the *L*. *barbarum* accessions in two groups derived from different breeding programs. Ningqi6 and Ningqi8 are bred by Ningxia Forestry Institute, and the other six *L*. *barbarum* accessions are bred by Ningxia Academy of Agriculture and Forestry sciences. *Lycium barbarum*, black fruit wolfberry (*L*. *ruthenicum* Murr), and big leaf wolfberry (*L*. *chinense* Mill), were divided into 3 different groups, reflecting their species differentiation. Korea wolfberry and big leaf wolfberry (*L*. *chinense* Mill) are in the same group, however, suggesting recent common ancestry. Further research with more accessions is needed to understand the genetic relationship among these two species. In general, the result supported the hypothesis that the EST-SSR markers described here are of good quality and can be used to evaluate genetic diversity efficiently. Therefore, the 22,537 deveoped EST-SSR markers provide a rich source of molecular markers that will facilitate genetic diversity analysis, genetic mapping and marker-assisted breeding in *L*. *barbarum*.

## Conclusion

The characterization of the *Lycium barbarum* transcriptome and the substantial body of transcripts obtained will facilitate investigations of its fruit development and its medicinal and nutritional components; and will also be of value to gene discovery and functional genomics studies. The SSR markers developed here provide a foundation for genetic diversity analysis, genetic mapping and marker-assisted breeding in *L*. *barbarum*.

## Supporting information

S1 Table Details of the primer pairs used for RT-qPCR. (XLSX)

S2 Table Details of the primer pairs used for PCR amplification. (XLS)

S3 Table Summary of the assembled unigenes in different databases. (RAR)

S4 Table Summary of the GO classification of assembled unigenes. (RAR)

S5 Table Summary of the KOG classification of assembled unigenes. (RAR)

S6 Table Summary of the KEGG classification of assembled unigenes. (RAR)

S7 Table Details of the 22,537 developed EST-SSR markers. (XLS)

S1 Fig Length distribution of transcripts. (TIF)

S2 Fig Length distribution of unigenes. (TIF)

S3 Fig Length distribution comparison of transcripts and unigenes. (TIF)
