# Supplementary figures and images for: Characterization of the Lycium barbarum fruit transcriptome and development of EST-SSR markers
Source: PLoS One. 2017 Nov 10;12(11):e0187738. doi: 10.1371/journal.pone.0187738 (PMC5695279; doi:10.1371/journal.pone.0187738)

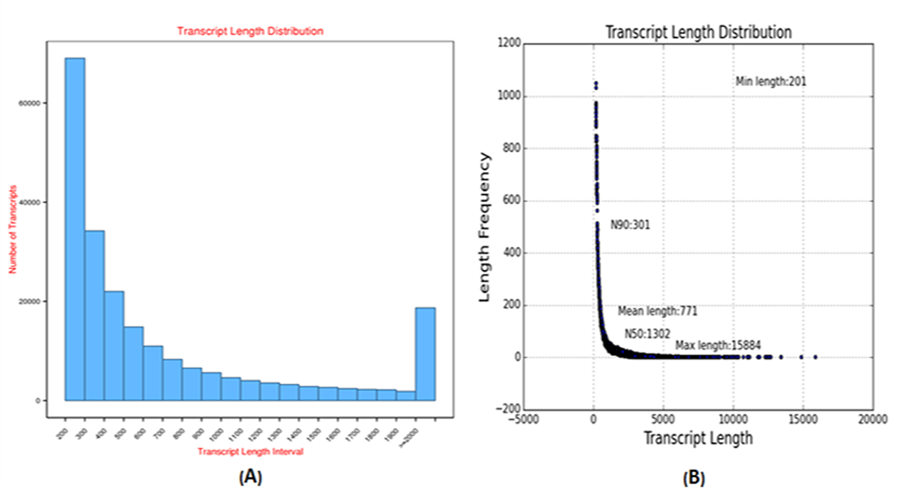

Supplement: S1 Fig — (TIF) [file pone.0187738.s008.tif]

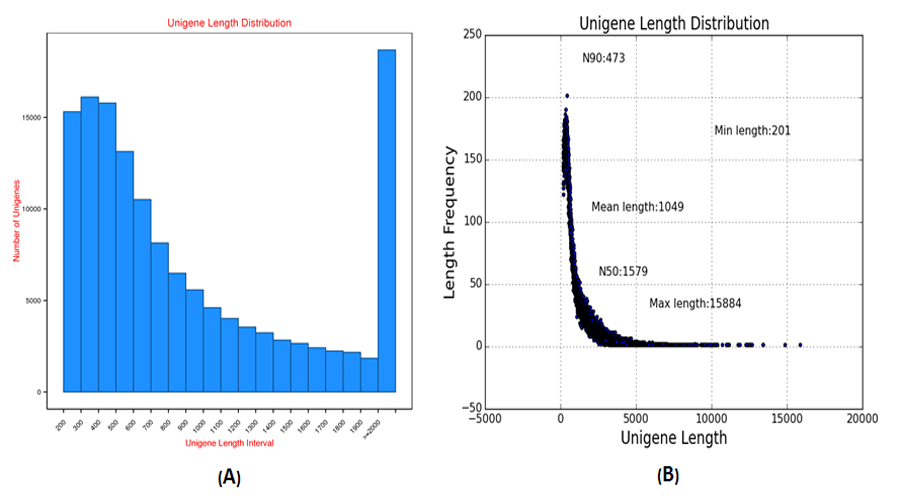

Supplement: S2 Fig — (TIF) [file pone.0187738.s009.tif]

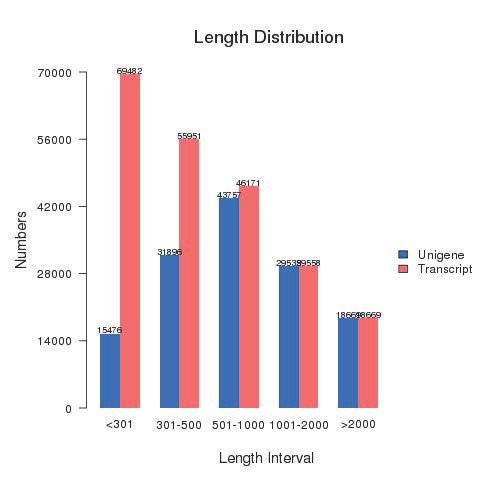

Supplement: S3 Fig — (TIF) [file pone.0187738.s010.tif]
